# Quantitative relationships between transforming growth factor beta mRNA isoforms in congenital and traumatic cataracts

**Published:** 2011-11-18

**Authors:** Pawel Banasiak, Barbara Strzalka-Mrozik, Maria Forminska-Kapuscik, Erita Filipek, Urszula Mazurek, Lidia Nawrocka, Ewa Pieczara, Piotr Berezowski, Malgorzata Kimsa

**Affiliations:** 1 Division of Pediatric Ophthalmology, Department of Ophthalmology, Medical University of Silesia, Katowice, Poland; 2 Department of Molecular Biology, Medical University of Silesia, Sosnowiec, Poland

## Abstract

**Purpose:**

The aim of this study was to determine differences in the expression profiles of transforming growth factor (*TGF*) β isoforms in the fragments of anterior lens capsules (ALCs) and peripheral blood mononuclear cells (PBMCs) of pediatric patients with congenital and traumatic cataracts.

**Methods:**

Forty children with congenital cataracts (19 girls and 21 boys) and 22 children with traumatic cataracts (six girls and 16 boys) participated in the study. Fragments of ALCs obtained during cataract surgery and whole blood samples were analyzed. Quantification of *TGFβ1*, *TGFβ2*, and *TGFβ3* mRNA was performed by real-time quantitative reverse transcription (QRT)-PCR using SYBR Green I chemistry.

**Results:**

*TGFβ1*, *TGFβ2*, and *TGFβ3* mRNA was detected in all the studied samples. Significant differences were found for *TGFβ1* and *TGFβ2* expression profiles in PBMCs between the patients with congenital and traumatic cataracts. The expression profiles of *TGFβ* isoforms in ALCs did not differ significantly between the groups.

**Conclusions:**

Overexpression of *TGFβ1* and *TGFβ2* in the PBMCs of patients with congenital cataracts might indicate that these cytokines are involved in the development of lens opacity.

## Introduction

Cataracts represent a degenerative disorder of the lens, and remain a leading cause of blindness worldwide [1]. However, the molecular mechanisms of the formation of congenital and traumatic cataracts are still poorly understood. The identification of molecular and genetic events involved in these diseases may be essential for the better understanding of cataracts, as well as for the development of diagnostic markers and novel treatment strategies.

Transforming growth factor (*TGF*) β is a strong candidate inducer of the epithelial–mesenchymal transition and extracellular matrix production, which characterize congenital and traumatic cataracts in the human lens [2,3]. In addition, the evidence from animal studies in vitro and in vivo supports the hypothesis of the cataractogenic potential of *TGFβ* [2,4-6].

Five members of the *TGFβ* family have been identified. However, only *TGFβ1*, *TGFβ2*, and *TGFβ3* have been demonstrated to be expressed in mammals [7]. Each isoform is encoded by unique genes of different chromosomal location [8], and reveals a 64–85% amino acid sequence homology [9]. All *TGFβ* isoforms have a similar biologic effect in vitro, but in vivo they are generally characterized by varied expression levels and different functions. Their biologic activity depends on quantitative relationships between individual isoforms [10,11].

Members of the *TGFβ* family regulate fundamental aspects of cellular functions, including cell growth, differentiation, inflammation, and wound healing [9-15]. Additionally, substantial evidence also implicates the role of *TGFβ* in many human diseases [2,4,11], including fibrotic diseases of the eye [5,6,16-18]. The relationship between *TGFβ* levels and a degree of fibrosis in various organs is well documented [19,20].

Most researchers determine only the TGFβ protein level using immunoenzymatic methods [20-26]. It should be mentioned that the change in the protein level is preceded by the alteration of gene transcriptional activity encoding this protein. Many attempts have been made to identify proteins in serum or mRNA in peripheral blood mononuclear cells (PBMCs), which could be easily accessed and act as markers of intratissue processes in various diseases [19,27]. However, there are no published data regarding differences between mRNA levels of all three *TGFβ* isoforms in the anterior lens capsules (ALCs) and PBMCs of pediatric patients with congenital and traumatic cataracts.

In the present study, real-time quantitative reverse transcription (QRT)-PCR was applied to investigate the changes in *TGFβ1*, *TGFβ2*, and *TGFβ3* gene expression in fragments of ALCs and PBMCs from pediatric patients with congenital and traumatic cataracts. Quantitative relationships between mRNA levels of these three *TGFβ* isoforms were analyzed.

## Methods

The patient group comprised 40 individuals (19 girls and 21 boys, mean age 9.8 years; range 4.7–17.3 years) with clinically diagnosed congenital cataracts. The comparison group consisted of 22 individuals (six girls and 16 boys, mean age 11.4; range 3.9–17.9 years) with clinically diagnosed traumatic cataracts (Table 1), treated in the Department of Ophthalmology, University Hospital No. 5, Medical University of Silesia, Katowice, Poland. The diagnosis of traumatic cataracts was based on the Birmingham Eye Trauma Terminology System [16]. The mean time interval between injury and cataract surgery was 14.9 months (range 0.2–156.2 months).

**Table 1:** Selected clinical features of the patients with clinically diagnosed congenital or traumatic cataracts.

**Characteristic**	**Congenital cataract (n=40)**	**Traumatic cataract (n=22)**
**Gender**		
F	19	6
M	21	16
Age (years)	9.8 (4.7–17.6)	11.3 (3.9–17.9)
**Eye**		
Right	18	9
Left	22	13
Mean time interval between injury and cataract surgery (months)	-	14.9 (0.2–156.2)
Mean age at the time of injury (years)	-	10.2 (3.7–17.6)
Type of trauma (BETTS*)		
Penetrating	-	22

Values of clinical parameters are expressed as means (minimum-maximum). Abbreviations M and F stand for male and female participants, respectively. *BETTS - Birmingham Eye Trauma Terminology System.

The criteria for inclusion in the molecular analysis were as follows: age ≤18 years, no inflammatory conditions (before the surgery, all children were examined by an anesthesiologist, pediatrician, ear, nose, and throat specialist, and dentist), and no systemic disease. In the case of children with congenital cataracts, we also excluded local disorders such as persistent hyaloid vessels, small cornea, and microphthalmia.

The study was approved by the Bioethics Committee of the Medical University in Katowice (KNW/0022/KB1/63/I/09) in accordance with the Declaration of Helsinki regarding medical research involving human subjects. The study and its purpose were explained to each participant or his or her legal guardian, who gave informed written consent.

### Tissues

Circular sections of ALCs with attached anterior lens epithelial cells were obtained during cataract surgery, and were stored for 48 h at −70 °C until RNA extraction. Venous blood samples were collected into EDTA-containing tubes, and a 7.5 ml sample from each patient was centrifuged on a Ficoll-Conray gradient (specific gravity 1.077; Immunobiological Co., Gumma, Japan) immediately after blood collection.

### Ribonucleic acid extraction from tissue specimens

Total RNA was extracted from PBMCs and from ALCs using TRIzol reagent (Invitrogen, Carlsbad, CA). RNA extracts were treated with DNase I (MBI Fermentas, Vilnius, Lithuania) according to the manufacturer’s instructions. The quality of extracts was checked electrophoretically using 0.8% agarose gel stained with ethidium bromide. The results were analyzed and recorded using the 1D Bas-Sys gel documentation system (Biotech-Fisher, Perth, Australia). Total RNA concentration was determined by spectrophotometric measurement in 5 μl capillary tubes using the Gene Quant II RNA/DNA Calculator (Pharmacia Biotech, Cambridge, UK).

### Real-time quantitative reverse transcription polymerase chain reaction assay

Gene expression of *TGFβ1*, *TGFβ2*, *TGFβ3*, and glyceraldehyde-3-phosphate dehydrogenase (*GAPDH*) genes were evaluated using real-time QRT–PCR and SYBR Green I chemistry (SYBR Green Quantitect RT–PCR Kit; QIAGEN, Valencia, CA). The analysis was performed using an Opticon™ DNA Engine Continuous Fluorescence Detector (MJ Research, Watertown, MA). All samples were tested in triplicate. *GAPDH* was included to monitor the QRT–PCR efficiency. Oligonucleotide primers specific for *TGFβ1*, *TGFβ2*, *TGFβ3*, and *GAPDH* were described previously by Strzalka et al. [9,15] and Ercolani et al. [28] (Table 2). The thermal profile for one-step RT–PCR was as follows: reverse transcription at 50 °C for 30 min, denaturation at 95 °C for 15 min, and 50 cycles consisting of temperatures: 94 °C for 15 s, 60 °C for 30 s, and 72 °C for 30 s. The point at which a PCR product is first detected above a fixed threshold, termed a cycle threshold (Ct), was determined for each sample, and an average Ct of triplicate samples was calculated. Each run was completed using melting curve analysis to confirm the specificity of amplification and the absence of primer dimers. RT–PCR products were separated on 6% polyacrylamide gels and visualized with silver salts.

**Table 2:** Characteristic of primers used for real-time QRT-PCR.

Gene	Sequence of primers	Length of amplicon (bp)	Tm (ºC)
*GAPDH*	Forward: 5’-GAAGGTGAAGGTCGGAGTC-3’	226	80
	Reverse: 5’-GAAGATGGTGATGGGATTC-3’		
*TGFβ1*	Forward:5’-TGAACCGGCCTTTCCTGCTTCTCATG-3’	151	85
	Reverse: 5’-GCGGAAGTCAATGTACAGCTGCCGC-3’		
*TGFβ2*	Forward: 5’-TACTACGCCAAGGAGGTTTACAAA-3’	201	80
	Reverse: 5’-TTGTTCAGGCACTCTGGCTTT-3’		
*TGFβ3*	Forward: 5’-CTGGATTGTGGTTCCATGCA-3’	121	81
	Reverse: 5’-TCCCCGAATGCCTCACAT-3’		

bp – base pairs; Tm -melting temperature.

### Quantification of expression of target genes

To quantify the results obtained by RT–PCR for *TGFβ1*, *TGFβ2*, *TGFβ3*, and *GAPDH*, a standard curve method was employed [9,15]. Commercially available standards of β-actin (*ACTB*) cDNA (TaqMan® DNA Template Reagent Kit; PE Applied Biosystems, Inc., Foster, CA) were used at five different concentrations (0.6, 1.2, 3.0, 6.0, and 12.0 ng/µl) to simultaneously detect the expression profile of each investigated gene. For standards, the calculation of copy number values was based on the following relationship: 1 ng of DNA=333 genome equivalents (PE Applied Biosystems). Amplification plots for each dilution of a commercially available standard template were used to determine Ct values [9,15]. A standard curve was generated by plotting Ct values against the log of the known amount of *ACTB* cDNA copy numbers. Correlation coefficients for standard curves ranged from 0.988 to 0.995, indicating a high degree of confidence for measurement of the copy number of molecules in each sample. The copy numbers of analyzed mRNAs were calculated from linear regression of the standard curve.

### Statistical analyses

Statistical analyses were performed using Statistica 8.0 software (StatSoft, Tulsa, OK), and the level of significance was set at p<0.05. Values were expressed as median (Me) with the 25th and 75th quartiles. Nonparametric tests were used for statistical analyses because the Shapiro-Wilk test indicated that the data were not normally distributed. The Kruskal–Wallis test and post hoc multiple test based on average ranks were applied to assess differences in the expression of *TGFβ* isoforms. Intergroup comparison of the gene expression under investigation was performed with the Mann–Whitney U test. Correlations were evaluated using the Spearman rank correlation test.

## Results

### Specificity of the real-time reverse transcription polymerase chain reaction assay

RT–PCR specificity for the target genes was confirmed experimentally on the basis of amplimers’ melting temperatures. For each RT–PCR product, a single peak at expected temperatures was observed: *TGFβ1*, 85.4 °C; *TGFβ2*, 80.0 °C; *TGFβ3*, 80.6 °C; and *GAPDH*, 80.1 °C (data not shown). Gel electrophoresis also revealed the presence of a single product of predicted length (data not shown).

### Differences in transforming growth factor β1, β2, and β3 messenger ribonucleic acid between anterior lens capsules and peripheral blood mononuclear cells

In this part of the study, the expression of *TGFβ1*, *TGFβ2*, and *TGFβ3* was analyzed by real-time QRT–PCR. Then, the quantitative relations between the mRNA of these three isoforms in congenital and traumatic cataracts were evaluated.

**Figure 1:**
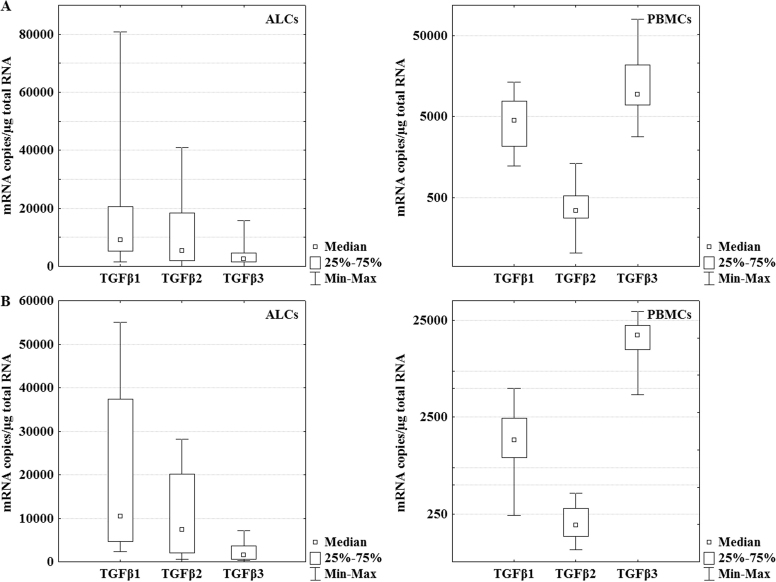
The mRNA levels of transforming growth factor β isoforms in anterior lens capsules and peripheral blood mononuclear cells of patients with clinically diagnosed congenital (**A**) and traumatic cataracts (**B**). Box and whisker plots present medians±quartiles and extreme values of copy numbers per 1 μg of total RNA; p<0.0001, Kruskal–Wallis test.

*TGFβ1*, *TGFβ2*, and *TGFβ3* isoforms were detected in ALC and PBMC samples obtained from patients with congenital and traumatic cataracts (Figure 1A,B).

A comparative analysis of all *TGFβ* mRNA copies/µg of total RNA revealed that *TGFβ1* was a predominant isoform in the ALCs of patients with congenital cataracts. There was no statistically significant difference between *TGFβ1* (Me=9162) and *TGFβ2* (Me=5427) mRNA levels (p=0.113, post hoc test). However, the mRNA level of *TGFβ3* (Me=2470) was significantly lower than those of *TGFβ1* (p<0.001, post hoc test) and *TGFβ2* (p<0.001, post hoc test). In the PBMCs of patients with congenital cataracts, the expression of *TGFβ3* (Me=9395) was the highest and significantly greater than those of *TGFβ1* (Me=4475, p=0.015, post hoc test) and *TGFβ2* (Me=343, p<0.001). The mRNA level of *TGFβ1* was also significantly greater than that of *TGFβ2* (p<0.001, post hoc test).

The quantitative relations between mRNA of these three isoforms were similar in the patients with clinically diagnosed traumatic cataracts. *TGFβ1* was also the main isoform in ALCs. The mRNA levels of *TGFβ1* (Me=10501) and *TGFβ2* (Me=7380) were not statistically significant (p=0.226, post hoc test). However, the expression of *TGFβ3* (Me=1573) was found to be significantly lower than those of *TGFβ1* (p<0.001, post hoc test) and *TGFβ2* (p=0.013, post hoc test) in ALCs. For PBMCs, the relationships were as follows: the *TGFβ3* mRNA level (Me=17650) was the highest and significantly greater than those of *TGFβ1* (Me=1459, p=0.002, post hoc test) and *TGFβ2* (Me=190, p=0.012, post hoc test). The mRNA level of *TGFβ1* was also significantly greater than that of *TGFβ2* (p<0.001, post hoc test).

### Correlations between transforming growth factor β1, β2, and β3 messenger ribonucleic acid for anterior lens capsules and peripheral blood mononuclear cells

All three isoforms were positively correlated with each other in the ALCs of patients with congenital cataracts. However, there was only a significant association between mRNA levels of *TGFβ1* and *TGFβ2* for PBMCs (Table 3).

**Table 3:** The correlations between *TGFβ* isoforms (copies/μg total RNA) in the ALCs and PBMCs of patients with clinically diagnosed congenital and traumatic cataracts.

**Congenital catarct**							
ALCs	*TGFβ1* mRNA	*TGFβ2* mRNA	*TGFβ3* mRNA	PBMCs	*TGFβ1* mRNA	*TGFβ2* mRNA	*TGFβ3* mRNA
*TGFβ1* mRNA	-	r=0.683*	r=0.596*	*TGFβ1 *mRNA	-	r=0.546*	r=0.213
*TGFβ2* mRNA	r=0.683*	-	r=0.569*	*TGFβ*2 mRNA	r=0.546*	-	r=-0.129
*TGFβ3* mRNA	r=0.596*	r=0.569*	-	*TGFβ3* mRNA	r=0.213	r=-0.129	-
**Traumatic cataract**							
ALCs	*TGFβ1* mRNA	*TGFβ2* mRNA	*TGFβ3* mRNA	PBMCs	*TGFβ1* mRNA	*TGFβ2* mRNA	*TGFβ3* mRNA
*TGFβ1* mRNA	-	r=0.583*	r=0.279	*TGFβ1* mRNA	-	r=-0.218	r=0.673*
*TGFβ2* mRNA	r=0.583*	-	r=0.331	*TGFβ2* mRNA	r=-0.218	-	r=-0.151
*TGFβ3* mRNA	r=0.279	r=0.331	-	*TGFβ3* mRNA	r=0.673*	r=-0.151	-

Statistical significance: *p<0.05, Spearman rank correlation test.

For traumatic cataracts, a positive correlation was only observed between the expression of *TGFβ1* and *TGFβ2* in ALCs, whereas for PBMCs, a positive correlation was only affirmed between the expression of *TGFβ1* and *TGFβ3* (Table 3).

### Differences in transforming growth factor β1, β2, and β3 messenger ribonucleic acid between congenital and traumatic cataracts

In ALCs, there was no significantly different expressions of *TGFβ1*, *TGFβ2*, and *TGFβ3* between congenital and traumatic cataracts (*TGFβ1* p=0.78, *TGFβ2* p=0.75, *TGFβ3* p=0.23, Mann–Whitney U test). In PBMCs, the expression of *TGFβ1* and *TGFβ2* was about twofold higher in the congenital cataract patients compared to the patients with traumatic cataracts, and a statistical significance was found (*TGFβ1* p<0.0001, *TGFβ2* p<0.0001, Mann–Whitney U test). However, no statistically significant relationship was found for *TGFβ3* isoforms (p=0.23, Mann–Whitney U test; compare Figure 1A and Figure 1B).

### Relationships between transforming growth factor β1, β2, and β3 messenger ribonucleic acid, gender, and left- or right-sided cataracts

No statistically significant correlations were found for congenital cataracts between the mRNA levels of *TGFβ* isoforms and gender or left- or right-sided cataracts (Table 4).

**Table 4:** Factors associated with *TGFβ* isoforms’ expression changes in the ALCs and PBMCs of patients with clinically diagnosed congenital cataracts.

**Variable**	**n**	TGFβ1*	p**	TGFβ2*	p**	TGFβ3*	p**
**Anterior lens capsules (ALCs)**							
**Gender**							
M	21	9682 (4554–19139)	NS	5119 (1967–15660)	NS	1915 (1494–2845)	NS
F	19	9162 (5792–24306)		8034 (2154–19968)		3082 (2062–8230)	
**Eye**							
Right	18	11122 (5790–15423)	NS	7161 (2518–18149)	NS	2524 (1564–5017)	NS
Left	22	8942 (4621–24306)		5119 (1967–18430)		2295 (1527–3888)	
**Peripheral blood mononuclear cells (PBMCs)**							
**Gender**							
M	21	3640 (1519–8691)	NS	356 (295–529)	NS	8998 (7261–15053)	NS
F	19	4475 (2749–5010)		330 (265–506)		16768 (4119–35775)	
**Eye**							
Right	18	4475 (1787–7761)	NS	356 (300–552)	NS	11827 (6327–35775)	NS
Left	22	4070 (2121–7105)		308 (266–513)		9788 (6943–19801)	

*Median with 25th and 75th quartiles (mRNA copies/µg total RNA). **U Mann–Whitney test. NS – not significant. Abbreviations M and F stand for male and female participants, respectively.

### Correlations between transforming growth factor β1, β2, and β3 messenger ribonucleic acid and patient’s age or time interval between trauma and surgery

There was no correlation between the age and transcriptional activity for any of the *TGFβ* isoforms in the ALCs of the congenital cataract samples collected during surgery (ALCs: *TGFβ1*, r=0.180, not significant (NS); *TGFβ2*, r=0.168, NS; *TGFβ3*, r=-0.011, NS). For PBMCs, a significant negative correlation was only determined between the mRNA levels of *TGFβ1* and age (PBMCs: *TGFβ1*, r=-0.395, p<0.05; *TGFβ2*, r=-0.025, NS; *TGFβ3*, r=-0.051, NS). Correspondingly to the results obtained for congenital cataracts, in ALCs from the patients with traumatic cataracts, no correlations between the expression of *TGFβ* genes and age (ALCs: *TGFβ1*, r=-0.158, NS; *TGFβ2*, r=0.110, NS; *TGFβ3*, r=-0.046, NS) were detected. As for PBMCs, a positive correlation was only observed between *TGFβ3* mRNA and age (PBMCs: *TGFβ3*, r=0.657, p<0.05; *TGFβ1*, r=-0.395, NS; *TGFβ2*, r=-0.042, NS).

Furthermore, correlations between *TGFβ1*, *TGFβ2*, and *TGFβ3* mRNA levels and time interval between trauma and surgery were evaluated in our study. There was a significant positive correlation only between the mRNA levels of *TGFβ1* and time interval between trauma and surgery in PBMCs (r=0.595, p<0.05).

## Discussion

*TGFβ* isoforms and their receptors are expressed in both normal and pathological lenses [2,18,29]. Determination of differences between mRNA levels in cataractous lens capsules compared and normal ones seems to be important. Previous research has revealed elevated levels of *TGFβ* in cataractous lenses in comparison to those in noncataractous ones [30]. Therefore, this report focused only on quantitative relations between *TGFβ* isoforms in congenital and traumatic cataracts. In the present study, real-time QRT–PCR was used to evaluate copy numbers of *TGFβ1*, *TGFβ2*, and *TGFβ3* mRNA. All three *TGFβ* isoforms were detected in ALC and PBMC samples, which is partially consistent with the results obtained by Gordon-Thomson et al. [2]. These authors demonstrated the presence of *TGFβ1* and *TGFβ2* mRNA in various ocular tissues, including the lens. *TGFβ3* mRNA was only detected in the retina and choroid. However, they observed mRNA distribution in ocular tissues using in situ hybridization methods.

Our results revealed that *TGFβ1* was a predominant isoform in ALCs, whereas *TGFβ3* was a major isoform in PBMCs in patients with congenital and traumatic cataracts. Our observation is supported by the data published by Lee et al. [30], who indicated the strongest expression of *TGFβ1* mRNA in human lens epithelial cells from patients with anterior polar cataracts. Similar results were demonstrated by Carrington et al. [31], who found *TGFβ1* to be a predominant isoform in the bovine cornea during wound healing. However, the real-time QRT–PCR technique was not used in these studies.

Shirai et al. [32] also performed studies where epithelial mesenchymal transition lens cells expressed *TGFβ1*, *TGFβ2*, and *TGFβ3* isoforms. This remains consistent with current results showing the presence of all three *TGFβ* isoforms in both types of cataracts. In ALCs, similar mRNA levels of predominant *TGFβ1* and *TGFβ2* isoforms were found. Whereas Shirai et al. [32] only determined the increase of both active and total TGFβ2 in the injured rat lens and aqueous humor, according to Yoneda et al. [33], the concentration of T*GFβ3* was much lower than the content of other two isoforms in the aqueous humor, which confirms our observations for ALC tissues.

Xiao et al. [34] revealed an overaccumulation of *TGFβ1* and basic fibroblast growth factor mRNA in the lens with anterior subcapsular congenital cataracts when compared to transparent lenses. In our study, it was observed that *TGFβ1* mRNA levels in ALC patients were raised, but there were no significant differences in its levels between congenital and traumatic cataracts, which partially corresponds to the research results of Xiao et al. [34]. The dissimilarity might have resulted from the selection of our control group, i.e., including pediatric patients who had sustained an eye injury rather than patients with clear lenses. The lack of significant differences between the two types of cataractous lenses suggests that *TGFβ* may mediate at least some part of cataractogenesis for both types.

Previous studies indicated that traumatic events could result in the activation of the *TGFβ* signaling pathway [17,18]. Wallentin et al. [35] demonstrated the activation of the *TGFβ* superfamily signaling pathway following experimental cataract surgery in rabbits. Furthermore, these authors showed that the level of total *TGFβ* in postoperative aqueous humor was continually increasing. Likewise, our results revealed higher mRNA levels of *TGFβ1* and *TGFβ2* in ALCs of traumatic cataract patients. However, these results were not statistically significant when compared to those obtained for congenital cataracts. The results shown above may indicate that cataract surgery increases the level of *TGFβ*s.

TGFβ has been found in human plasma, platelets, and circulating leukocytes [36-38]. It is known that the expression of *TGFβ* is constitutive in leukocytes and does not depend on activation [36]. Therefore, it seems a proper biologic material for the determination of TGFβ levels in blood. Our results for the quantitative relationships between three *TGFβ* isoforms in PBMCs are in accordance with other studies’ results [38]. Interestingly, the subjects with congenital cataracts showed a significant elevation of *TGFβ1* and *TGFβ2* expression in PBMCs when compared to those with traumatic cataracts, which suggests that traumatic cataractogenesis is mediated differently from congenital cataractogenesis. The most striking difference concerned *TGFβ1*. Chan et al. [39] studied the plasma level of the *TGFβ1* transgene in hybrid mice. Although the expression of the *TGFβ1* transgene was only targeted to the liver, its overexpression resulted in hepatic and multiple extrahepatic lesions. In the eye, there was a progressive cortical cataract formation. In our research, the upregulation of *TGFβ1* and *TGFβ2* gene expression in the PBMCs of patients with congenital cataracts might suggest that the processes occurring in the lens are affected by systemic expression levels of these cytokines, especially during fetal life when the lens is vascularized.

The second part of this study focused on the correlations between *TGF* isoforms in congenital and traumatic cataracts. Interestingly, in the ALCs of congenital cataract patients, all three *TGFβ* isoforms were positively correlated, but in traumatic cataract patients, there were only positive correlations between the mRNA levels of *TGFβ1* and *TGFβ2*. Moreover, in the PBMCs of congenital cataract patients, only *TGFβ1* and *TGFβ2* exhibited positive association, whereas in the traumatic cataract patients, the *TGFβ1* and *TGFβ3* isoforms were correlated. The relations between the levels of *TGFβ1*, *TGFβ2*, and *TGFβ3* and their biologic effects may be explained through TGFβ binding to the three cell surface receptors. Although *TGFβ* isoforms share the same receptors for their signal transduction, a specific pathological role played by *TGFβ*s might result from their different activity or potency [33].

In the studies performed by Rostkowska-Nadolska et al. [40], *TGFβ1* transcriptional activity was accompanied by *TGFβ2* transcriptional activity in nasal polyps, which is in accordance with the observations of their activity in the lenses of our study. The TGFβ2 isoform is structurally similar to TGFβ1, but the biologic responses to these cytokines differ depending on the cell type. The promoter sequences of the genes coding different *TGFβ* isoforms are not homologous. The analysis of *TGFβ1* and *TGFβ3* promoters revealed the presence of several binding sites for the transcriptional factors Sp-1 and AP-1. Fragments of the *TGFβ1* and *TGFβ3* promoters may compete for binding of Sp-1 to DNA. The *TGFβ2* promoter does not contain binding sites for the Sp-1 transcriptional factor [41]. The lack of correlation between *TGFβ*s may result from similar regulation of the expressions of these genes and competition for binding Sp-1.

Our data regarding congenital cataract revealed age-dependent differences in the mRNA levels of *TGFβ1* in PBMCs. These observations correspond to those of other researchers, who demonstrated that serum TGFβ1 levels tend to decrease with age [24,25]. However, Rungger-Brändle et al. [42] did not show any correlation between the *TGFβ2* mRNA levels and the age of the donors of the lenses without visible opacity, lenses with mature cataracts, and cataractous lenses with posterior subcapsular opacity or anterior subcapsular fibrosis, which is consistent with our results. On the other hand, it was found that rodent lens cells showed enhanced *TGFβ* receptors expression and increased susceptibility to the cataractogenic effects of *TGFβ* with age. This may suggest that the age-dependent increase in TGFβ signaling is connected with the responsiveness of the target tissue rather than with *TGFβ* expression itself [43,44].

Correlations between TGFβ2 levels and history of cataract surgery (time interval between previous cataract surgery and the evaluation of TGFβ2 concentrations) were observed in aqueous humor [45]. Taking into account Wallentin’s results [35], which show a continuous increase in total TGFβ after cataract surgery throughout the study period (30 days), the correlations between *TGFβ* gene expression and the time interval between trauma and surgery were evaluated in our studies. Our results revealed similar positive correlations between *TGFβ1* mRNA level and time interval between trauma and surgery for PBMCs. These observations might suggest that the accumulation of TGFβ promotes cataractogenesis.

At present, it is difficult to determine whether systemic *TGFβ1* and *TGFβ2* overexpression in congenital cataracts might act as a causative or concomitant factor. For instance, Marfan syndrome is believed to result from mutations in the gene encoding fibrillin-1. However, recent research has shed new light on the role of *TGFβ* in the pathogenesis of this disease. Interference of TGFβ signaling has been found to be involved in aortic aneurysm formation and progression. What is more, the disruption of *TGFβ* pathways might provide new diagnostic and therapeutic options [46,47]. Perturbations of TGFβ signaling might also underlie some proportion of congenital cataracts. Further studies are needed to conclusively confirm the cataractogenic influence of TGFβ.

In conclusion, all three *TGFβ* isoforms were found to be differentially expressed in the ALCs and PBMCs of pediatric patients with congenital and traumatic cataracts. Overexpression of *TGFβ1* and *TGFβ2* in the PBMCs of patients with congenital cataracts might indicate the involvement of these cytokines in the development of lens opacity.
